# What is the difference between perceived and actual risk of distracted driving? A field study on a real highway

**DOI:** 10.1371/journal.pone.0231151

**Published:** 2020-04-02

**Authors:** Zhen Li, Chang Wang, Rui Fu, Qinyu Sun, Hongjia Zhang

**Affiliations:** School of Automobile, Chang'an University, Xi'an, Shaanxi, China; University of British Columbia, CANADA

## Abstract

Distracted driving is a leading cause of traffic accidents. It is influenced by driver attitude toward secondary tasks; however, field-based studies on the effects of low-perceived-risk tasks on lateral driving have rarely been reported. A total of 17 experienced non-professional drivers were recruited to participate in two secondary tasks: a cognitive experiment (conversation) and a visual distraction experiment (observation of following vehicles), each representing low-perceived-risk secondary tasks. One-way analysis of variance (ANOVA) was conducted to evaluate the effects of low-perceived-risk tasks on lateral driving performance. ANOVA results indicated that compared with baseline (no task) lateral performance, lane-keeping ability was enhanced during cognitive distractions. In the visual distraction experiment, more than 50% of the distractions required 1–2 s. Lane deviation and its growth rate increased with the duration of distraction. Compared with cognitive distraction, lane deviation increased significantly with visual distraction, and lane-keeping performance was seriously impaired. For low-perceived-risk tasks, visual distractions impaired driving safety more seriously, compared with cognitive distractions, suggesting that drivers misjudge the risks associated with visual tasks. These results can contribute to the design of advanced driving-assistance systems and improve professional driver programs, potentially reducing the frequency of traffic accidents caused by distracted driving.

## 1. Introduction

Distracted driving due to secondary tasks is the main cause of traffic accidents. These secondary tasks include cognitive tasks (e.g., conversation and texting) and visual tasks (e.g., attending to surrounding vehicles and billboards) [1–3]. Driving distractions reduce the lane-keeping performance of drivers, potentially leading to serious traffic crashes [4, 5]. In the United States, distracted driving caused 3,477 deaths in 2015, 14.5% of which were attributed to unstable lateral vehicle control [6, 7].

Dangerous driving behaviors, including drunk driving and drowsy driving, have been prohibited by transportation laws and regulations in many countries. Use of mobile phones as a high-risk distraction task, has also been restricted in China since 2013; while driving, drivers are not allowed to hold a phone and only use Bluetooth or headsets to make calls [8]. However, in addition to using mobile phones, various secondary tasks can cause distracted driving [1, 4, 9], which cannot be completely avoided by merely applying laws. These distractions impair driving performance, and numerous studies have been conducted to categorize driving distractions. Cognitive distractions, visual distractions, and manual distractions comprise a widely used classification method [10]. Cognitive distractions occur when the driver is not entirely focused on the road (e.g., talking to passengers); visual distractions occur when the driver is distracted from keeping his or her eyes on the road (e.g., when concerned with the roadside environment or the rearview mirror); and manual distractions occur when the driver is distracted while operating certain devices (e.g., adjusting the radio).

In addition to legal constraints, driver self-regulation can effectively reduce the influence of distracted driving [11]. Many drivers have reported that they often change their driving behavior during secondary tasks, such as reducing their speed or stopping the car [12]. Oviedo–Trespalacios et al. and Wandtner et al. [13, 14] observed that drivers tend to decelerate while engaging in high-risk secondary tasks in a driving simulation experiment. Stavrinos et al. [15] found that drivers prefer to select various safe speeds in different driving scenarios. Wang et al. [16] indicated that drivers reduce their distraction duration while driving under high-risk conditions.

In self-regulation, crash risk perception plays a critical role, directly influencing driver attitude toward their level of self-regulation and road safety [17, 18]. Questionnaires have been used to investigate the subjective assessment by drivers of the risk associated with different distraction tasks. Patel et al. [19] distributed a subjective ranking survey that identified mobile phone use and makeup application as the riskiest behaviors. Meanwhile, the perceived risks associated with conversation and observing road signs or landscapes were low. Findings also suggest that drivers may not be more careful while engaging in conversation or observing driving scenarios. Gentzler et al. [20] similarly reported that conversation with passengers and looking at vehicles on the roadside were rated as low-risk distraction tasks by drivers. However, scenarios such as conversations and observing driving behaviors were common behaviors on actual roads [21, 22], classified as cognitive and visual distractions, respectively.

Despite these findings, studies on whether perceived secondary tasks are low-risk during actual driving are rarely reported. In a previous study, some experienced drivers considered performing a secondary task to constitute a safe driving behavior; that is, they could perform secondary tasks while driving safely [23]. However, excessive self-confidence in driving skills may reduce driver self-regulation in performing secondary tasks [24], affecting driving performance and increasing the likelihood of an accident.

Several driving metrics have been applied to evaluate the effects of distraction on driving performance. Steering wheel reversal rate (SRR) is a useful indicator for detecting visual and cognitive distraction. Kountouriotis et al. [25, 26] found that SRR increased during visual and cognitive distraction. In driving simulation experiments, the standard deviation of lane position (SDLP) was used to evaluate the lane-keeping performance of drivers during secondary tasks; research revealed that SDLP increased when dialing a mobile phone but decreased during a conversation [27, 28]. To evaluate the lane-keeping performance of the driver and the lateral position of the vehicle, lane deviation was considered as an intuitive indicator [29–31]. Despite numerous studies regarding the effects of driving distraction on lateral driving performance, research on the influence of driving duration on driving performance have rarely been reported. Ostlund et al. [32] demonstrated that task duration may influence lateral deviation; for the same driving task, the longer the time window duration, the greater the lane deviation. Thus, comparing the effects of different distraction tasks on lateral driving performance requires the selection of a fixed time or reference to the rate of lane deviation.

To examine the difference between perceived and actual risks associated with secondary tasks, this study evaluated the effects of low-perceived-risk secondary tasks (i.e., cognitive and visual distractions) on the lane-keeping performance of the driver on a real highway. Information on the lane position of the vehicle was collected by a lane recognition system installed in the test vehicle, and a front camera was used to record the visual distraction time. The lane position of the vehicle was used to evaluate the lateral control of the driver while performing cognitive and visual distraction tasks (conversation and observing the road scenarios, respectively). To compare two low-perceived-risk secondary tasks, a lane deviation rate was selected; analysis of variance (ANOVA) tests were performed to evaluate the effects of two types of distraction.

## 2. Methods

This section describes three field trials to investigate the effects of two low-perceived-risk secondary tasks on the lane-keeping ability of the driver. Two tasks—conversation and rear-vehicle observation—were selected as cognitive and visual tasks, respectively. Drivers considered each task to be of low perceived risk; these tasks represented the most common distractions on real roads [19, 20, 22]. All trials were performed on the same highway by using an instrumented vehicle.

### 2.1 Apparatus

**Fig 1:**
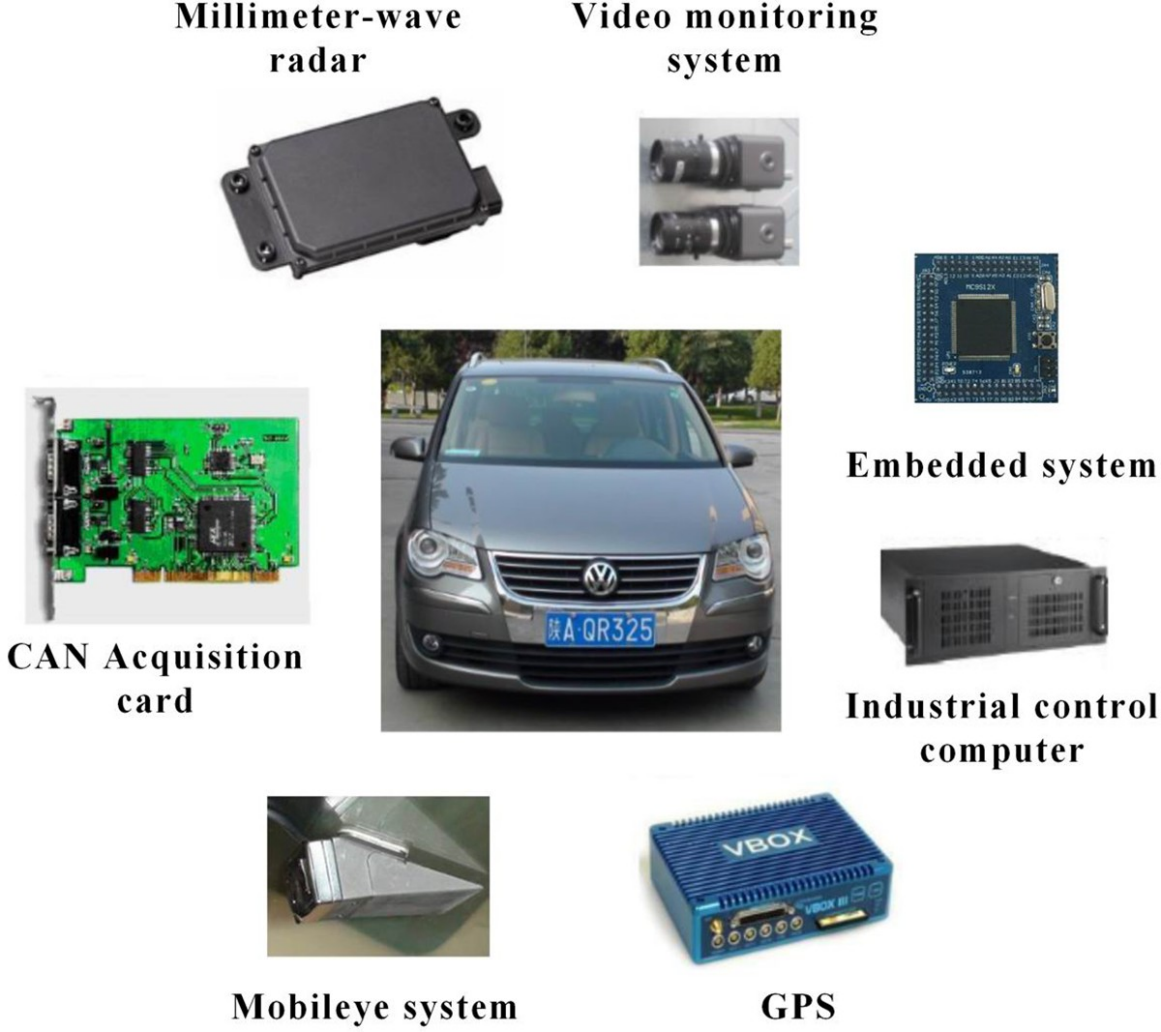
Instrumented vehicle.

As shown in Fig 1, the high-fidelity test platform is a real vehicle: the 2008 Volkswagen Touran equipped with a lane mark recognition system (Mobileye C2-170) and multiple high-speed cameras. The lane mark recognition system measured the distance of the test vehicle to the left and right lane marks at a system frequency of 10 Hz and with an accuracy of 5 cm. Multiple high-speed cameras were used to monitor driver head motion, driving behavior, and traffic environment.

### 2.2 Participants

Seventeen experienced drivers (15 males and 2 females) were recruited through an advertisement in Xi’an, China and were compensated for their participation. Each of the participants, aged 27–48 years (M = 34.7, SD = 7.7), had a valid driver’s license, normal or corrected-to-normal vision, and driving experience for 3–23 years (M = 8.4, SD = 5.2). None had been involved in a traffic accident during the past 3 years. The participants, all non-professional drivers, performed two driving tasks on the same road in the same car. Each driver had about 30 min of rest between driving trials.

### 2.3 Driving route

The drivers were asked to drive on the G3001 highway from Sanqiao to Xinzhu, a two-way, six-lane highway with a speed limit of 100 km/h. The route was 38 km long to allow participants to drive for roughly 30 min. Three trials were performed during nonpeak hours without traffic congestion. All tests were conducted under clear weather conditions to avoid any negative effect of weather on driver performance.

To prevent crashes, an experimental staff member with more than 20 years of driving experience was seated in the front passenger seat. The task of this staff member was to focus on the driving environment during the experiment. He was expected to inform the participant during the secondary task when a potential risk arose. The research team purchased insurance for all participants.

### 2.4 Cognitive distraction

Trial 1 examined the effect of a cognitive distraction task on the lane-keeping ability of the driver. In this trial, participants were required to perform a conversation task, which is considered a low-risk distraction on the basis of a subjective ranking of distraction tests. An experimental staff member sat in the rear passenger seat, and participants communicated with the staff member by answering a series of calculation questions. Lane deviation were recorded to analyze the effects of cognitive distractions.

Makishita [33] indicated that performing calculations can be considered as a serious conversation. In this trial, drivers were subjected to calculation tasks involving addition and subtraction of two positive two-digit numbers. In the addition task, the sum of the calculation was smaller than 100; in the subtraction task, the difference was not zero, and the answer could be either negative (e.g., 14 − 26 = -12) or positive (42 − 19 = 23). The drivers had to immediately answer orally the questions asked by the experimental staff member. If the participant responded correctly, the next question was asked; otherwise, the participant was again asked the same question. The staff member then asked the next question regardless of the correctness of the second answer.

### 2.5 Visual distraction

In Trial 2, we evaluated the effects of a visual distraction task on the lane-keeping ability of the driver. Before the test was conducted, participants were told that while driving, they would be estimating the relative distance and speed of a rear vehicle during the experiment, with no other requirements. Rear vehicles randomly approached the test vehicle and were not under experimental controls. Drivers were allowed to maintain their own driving styles and to avoid communicating with the experimental staff. In this test, each driver was asked to observe a rear vehicle through a rearview mirror whenever he/she felt doing so was safe. The driver was required to quickly estimate the rear-vehicle speed and relative distance as the rear vehicle approached the subject vehicle. Moreover, the driver orally reported this information to the staff.

**Fig 2:**
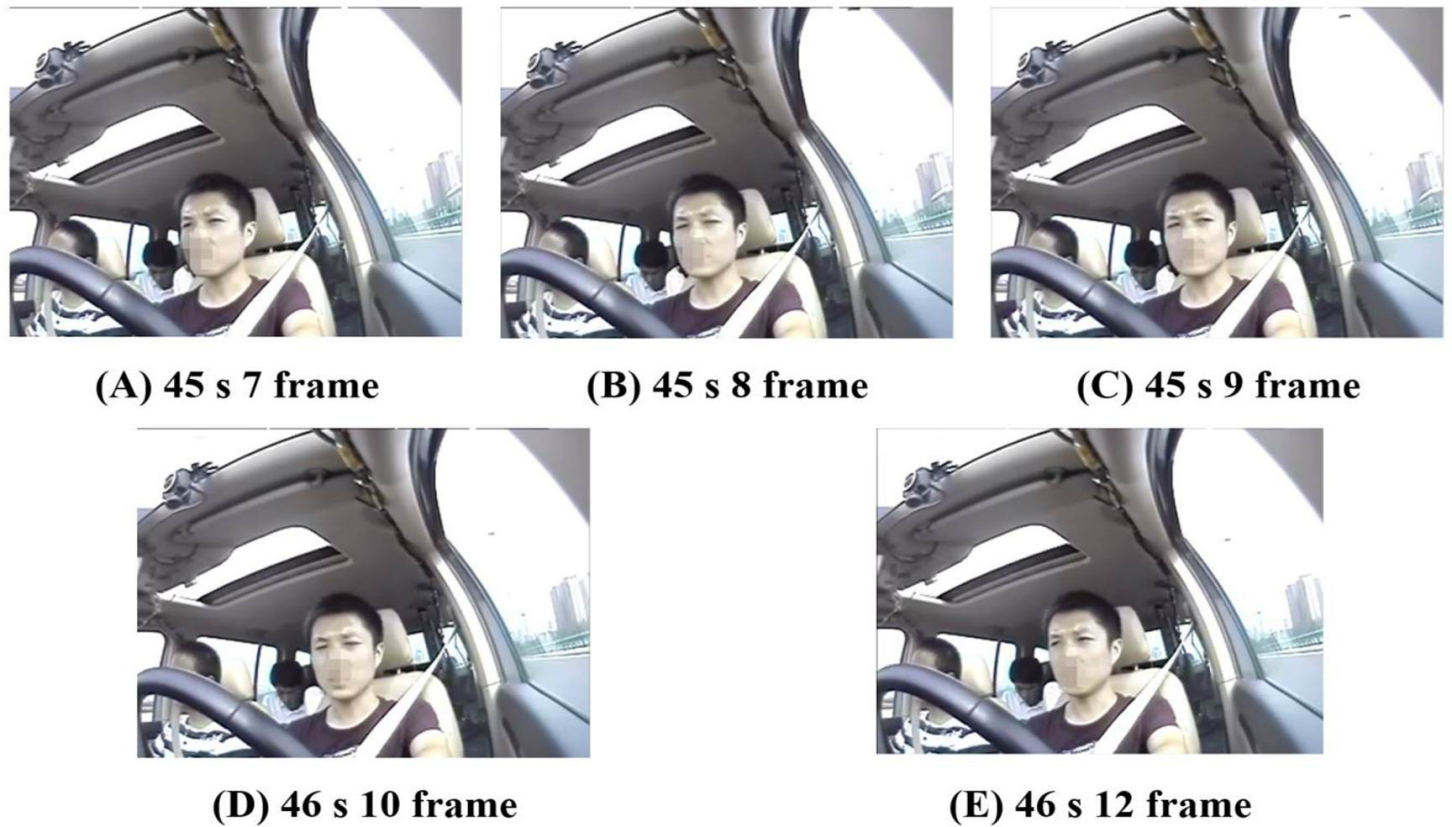
Driver distraction.

We distinguished the distraction time from his/her head motion and eye movement, as shown in the surveillance video. The start time was the moment the driving sight turned from the front to the rearview mirror, whereas the end time was the moment the driving sight turned from the rearview mirror to the front. Fig 2(A) shows driver sight and head position without visual distractions. Fig 2(B) shows the moment a driver checks the rearview mirror. Fig 2(C) shows the driver sight focused on the left-side rearview mirror. Fig 2(D) shows the moment the driver turns to look to the front. Fig 2(E) shows the driver sight returning to the front after visual distraction. The video consisted of 24 frames/s. Fig 2(B)–2(D) display 1 s of the time captured in 1 frame (the distraction duration is 1.042 s).

### 2.6 Normal driving

Driving behavior during normal driving was explored in this trial. Before the trial, the participants were required to drive the instrumented vehicle, using their typical driving style, on the test road for 30 min. The participants were not given any specific requirements when driving normally.

### 2.7 Lane deviation

Lane deviation, the difference in the lateral position of the vehicle at the starting and end moments of the task, was used to evaluate the lane-keeping ability of the drivers. On the basis of [34], the lateral position of a vehicle represents the distance between the center of the vehicle and the center lane mark.

**Fig 3:**
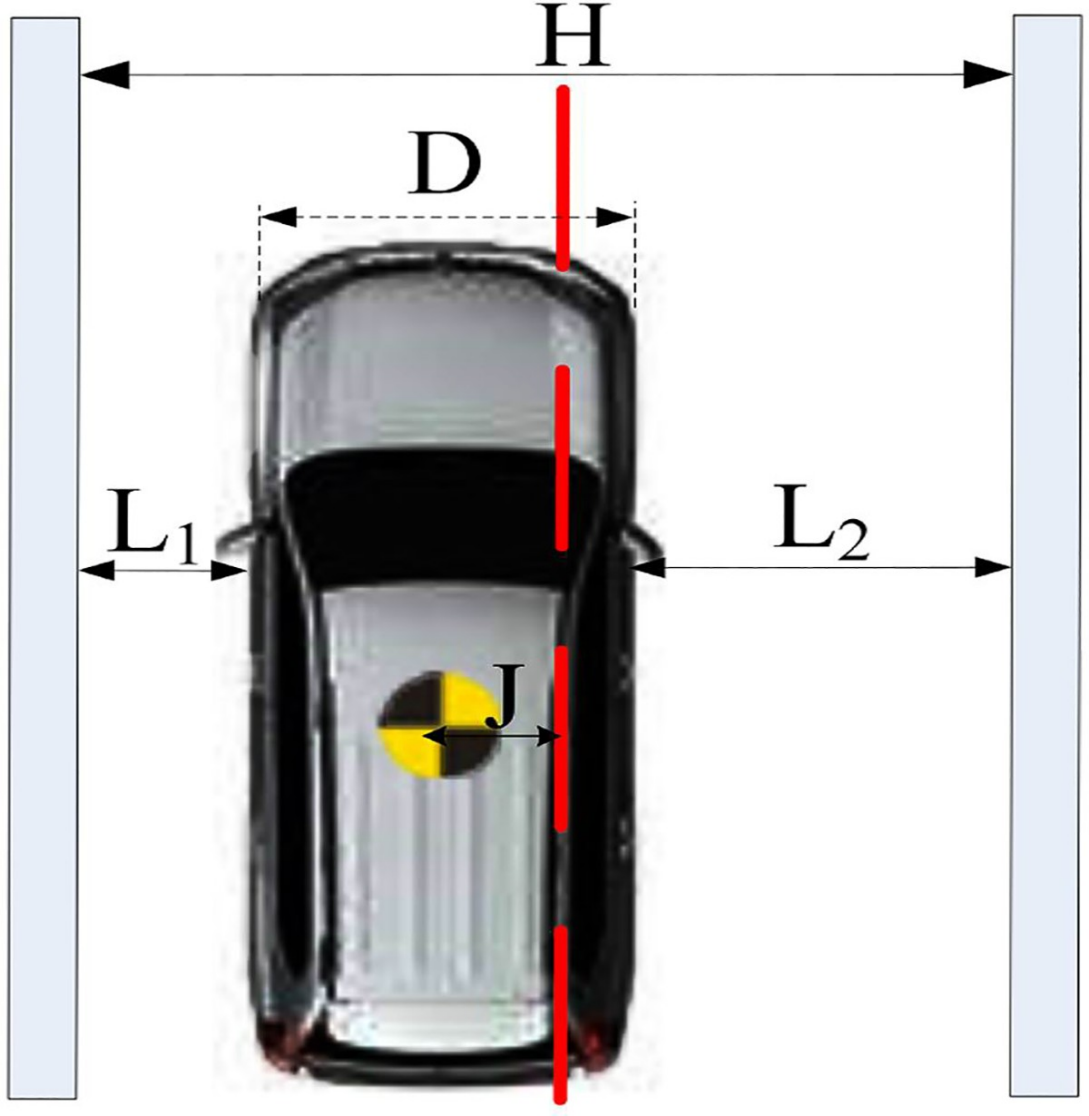
Lane position of the vehicle.

The lateral position of the vehicle is presented in Fig 3, where *H* is the lane width, *L*_*1*_ is the distance from the left lane to the left side of the vehicle, *L*_*2*_ is the distance from the right side of the vehicle to the right lane, *D* is the width of the vehicle, and *J* is the distance from the lane center to the center of the vehicle.

Vehicle lane-keeping characteristics can be represented by *J*. The formula to calculate *J* is shown in Eq (1):

$$J_{i}=\pm \frac{1}{2}|L_{1i}-L_{2i}|$$ (1)

where *L*_*1i*_ and *L*_*2i*_ denote the distance from the left and right lines (cm) at any time *i*, respectively; and ± represents the direction relative to the lane center (i.e., left is denoted by +, and right is denoted by −); and *J*_*i*_ is the vehicle lane position at any time *i*.

The lane deviation during a driving task can be calculated as

$$LD=|J_{2}-J_{1}|$$ (2)

where *LD* is the lane deviation (cm), *J*_1_ is the lane position at the start moment of the driving task, and *J*_2_ is the lane position at the end moment of the driving task. The rate of lane deviation was considered as the mean lane deviation during a driving task.

### 2.8 Procedure

Before the experiment, each driver completed a preparation phase of adaptive driving on a test road. Participants informed the staff once they had adapted to the experimental vehicle and the test road. Subsequently, the experiment officially began. The entire driving procedure consisted of three stages: the normal driving stage, cognitive distraction stage, and visual distraction stage. These three trials were performed at random with 15 min intervals.

In the cognitive distraction stage, the participants were asked questions continuously throughout the driving task, and the experimental staff recorded the answers. After the experiment, the driving status of the drivers was distinguished based on the recorded time and surveillance video. Information on lane departure was obtained by processing the data collected using the lane mark recognition system. In the visual distraction stage, the staff indicated when a rear vehicle was approaching, and the participant needed to quickly report the relative distance and driving speed of the rear vehicle.

### 2.9 Ethics and authorization statement

The experimental protocol was approved by the research committee of Chang’an University. Informed consent was obtained from each participant.

No permits were required for the described study, which complied with all relevant regulations.

## 3. Result

**Fig 4:**
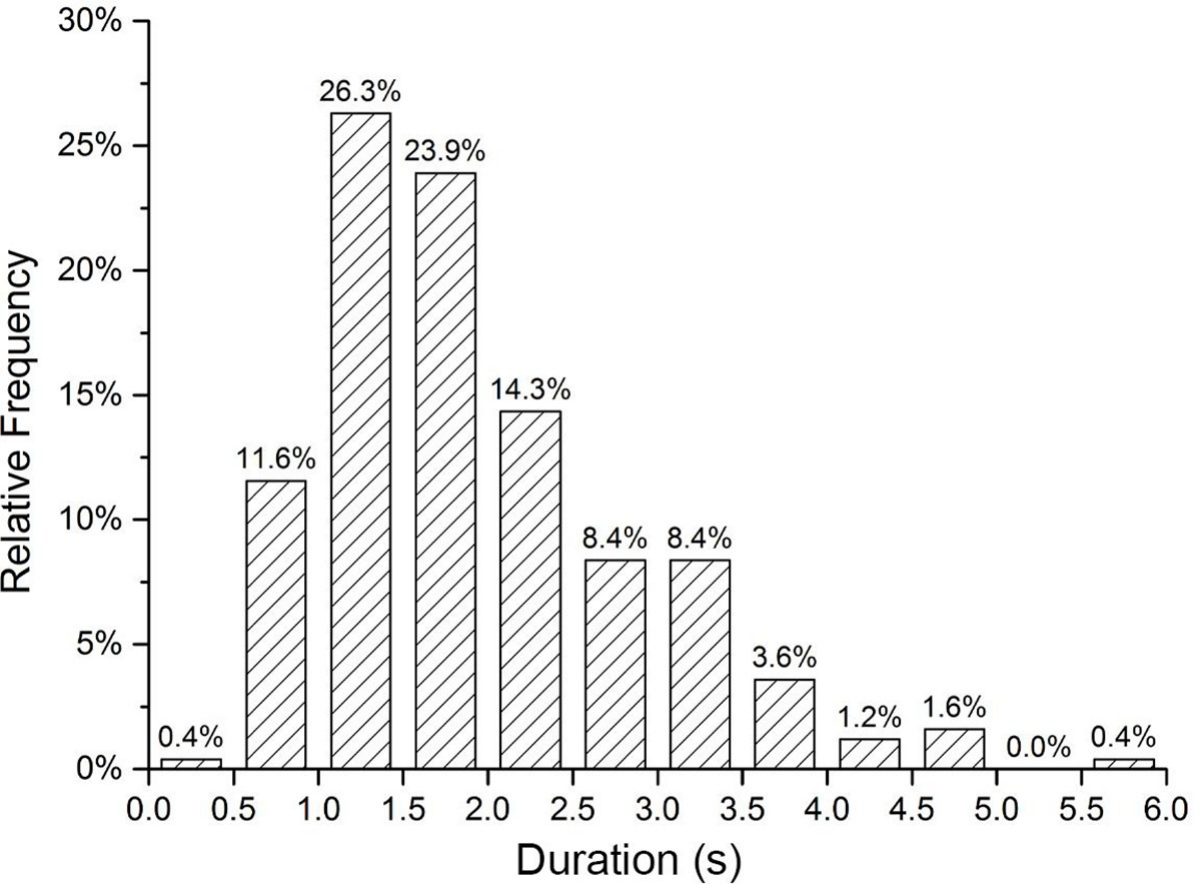
Distraction duration distribution.

A total of 247 visual distraction segments were recorded. The minimum visual distraction duration was 0.13 s, the maximum duration was 5.63 s, the average duration was 1.90 s, and the standard deviation was 0.94 s. The distribution of visual distractions is presented in Fig 4.

Among the visual distraction segments, the most frequent distraction duration was in the 1.0–1.5 s range. The distraction duration of 66 visual distraction segments was in the 1.0–1.5 s range, which comprising 26.3% of the total duration. When the distraction duration was shorter than 1.0 s, the duration was positively correlated with the number of distraction segments; when the distraction time was longer than 1.5 s, the distraction duration was negatively correlated with the number of distraction segments. The visual distraction duration mainly ranged within 1.0–2.0 s, constituting 50.2% of the total distraction time. More than nine-tenths of the visual distraction tasks had a duration of 0.5–3.5 s.

**Fig 5:**
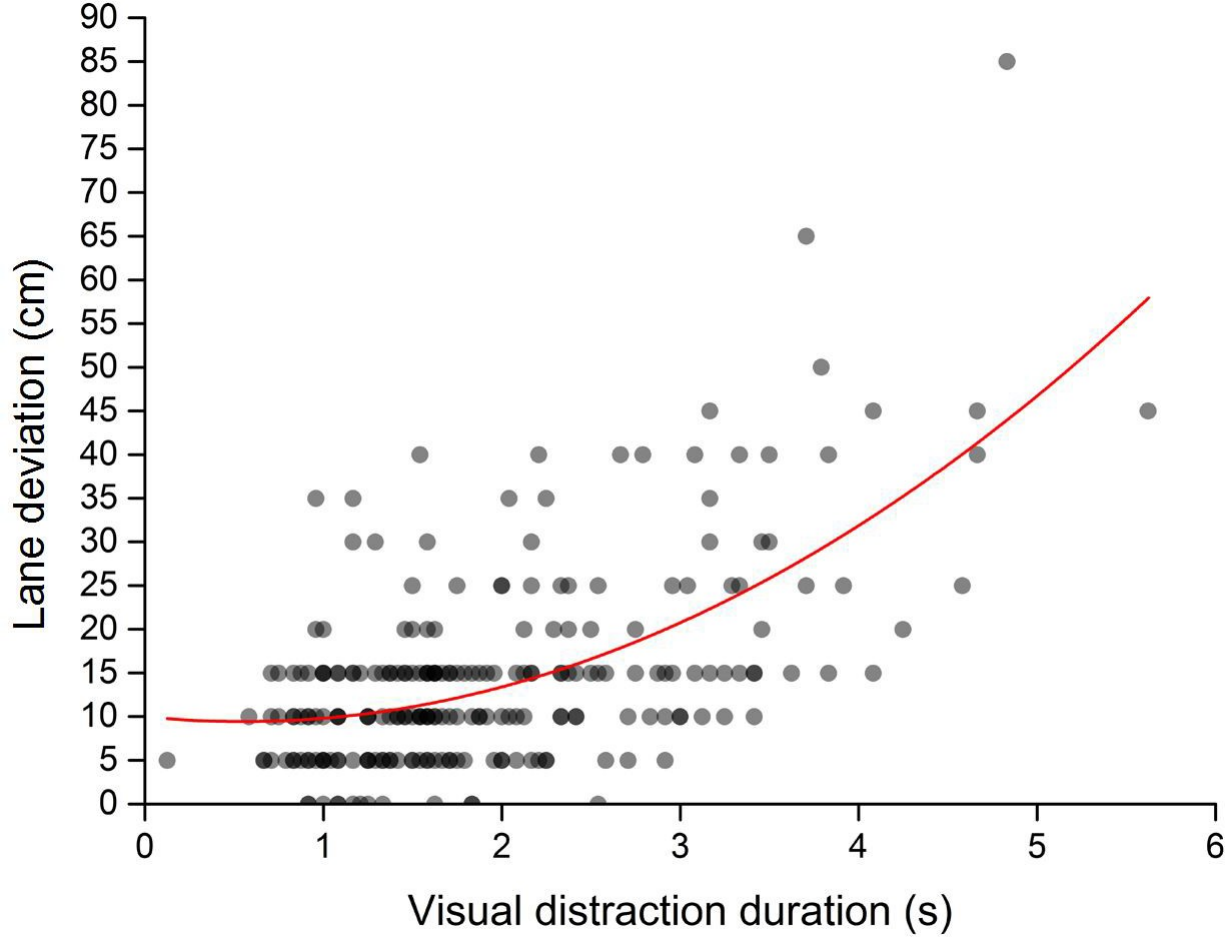
Quadratic regression of lane deviation and distraction duration.

**Fig 6:**
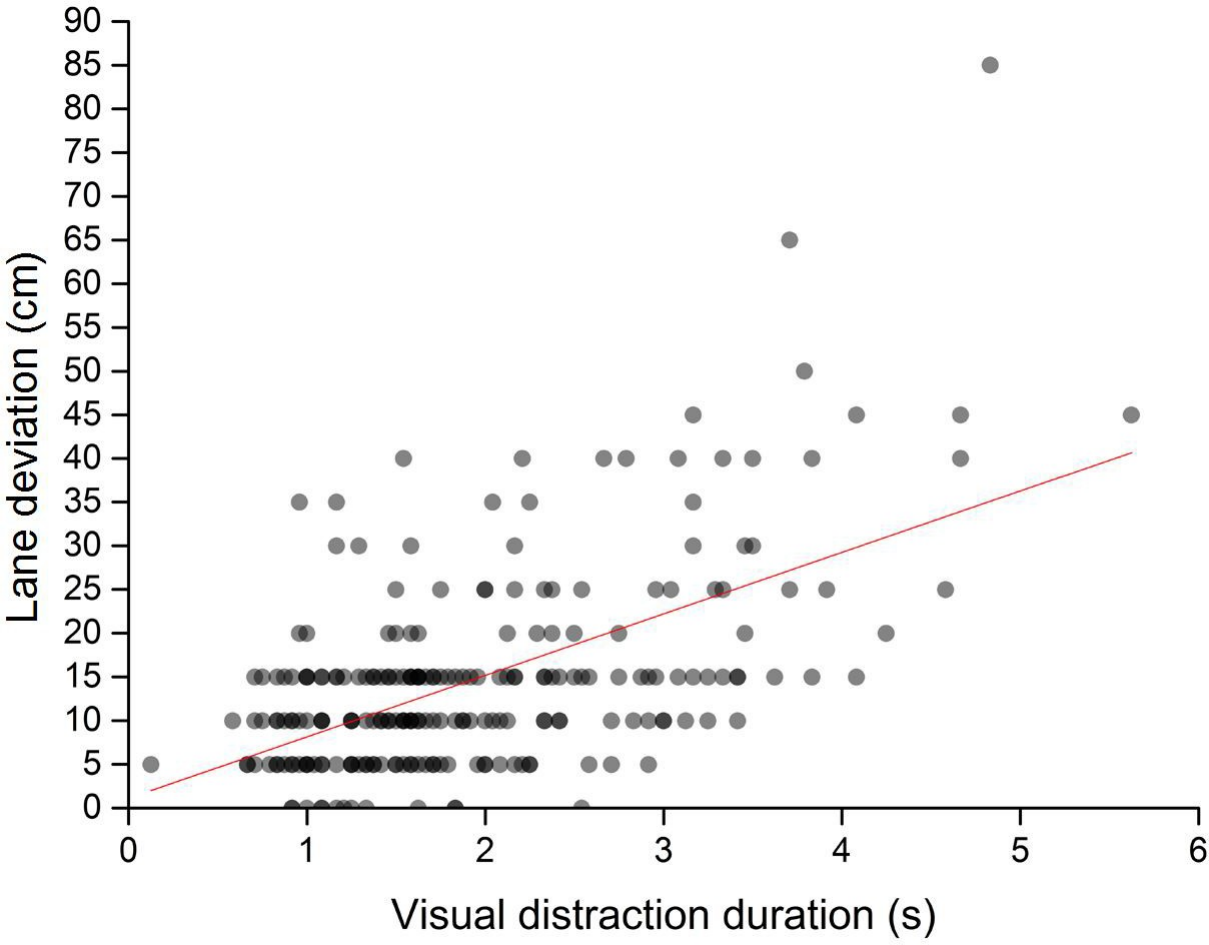
Linear fitting of lane deviation and distraction duration.

In assessing the trend of change in lane deviation with distraction duration, we used quadratic regression and linear regression to establish regression equations for lane deviation. The quadratic regression curve and scatterplot of lane deviation and distraction duration are presented in Fig 5. The fit statistics for this model indicated that quadratic regression was a good fit to the data (*p* < 0.001). The linear regression and scatterplot of lane deviation and distraction duration are presented in Fig 6. The fit statistics for this model indicated that linear regression was a good fit to the data (*p* < 0.001). Compared with linear fitting (R^2^ = 0.326), the quadratic regression (R^2^ = 0.363) were better. The comparison results showed that the change trend of lane deviation with the distraction duration is more in line with the quadratic change, suggesting that the growth rate of the lane deviation increases with an increase in distraction duration and that the safety of the vehicle decreases rapidly.

Owing to the low number of partial-interval segments, we divided the distraction process into seven ranges and conducted ANOVA on lane deviation in SPSS. The main effect of distraction duration was significant, F(6, 240) = 16.326, *p* < 0.001 (Table 1). A post-hoc test revealed no significant difference in lane deviation between distraction durations in the [0.0, 1.0), [1.0, 1.5), and [1.5, 2.0) ranges. No significant difference in lane deviation was found between distraction durations in the [1.5, 2.0), [2.0, 2.5), and [2.5, 3.0) ranges. The lane deviations (15.69 and 15.95 cm) were significantly higher when the distraction duration were in the [2.0, 2.5), and [2.5, 3.0) than the lane deviations (9.50 cm, 10.30 cm) when the distraction duration were in the [0.0, 1.0) and [1.0, 1.5) ranges. Moreover, the lane deviation was 36.76 cm when the distraction duration was in the [3.5, 6.0) range, which is significantly higher than those in the other six time intervals. The lane deviation was 21.67 cm when the distraction duration was in the [3.0, 3.5) range; a significant difference from lane deviations in the other six time intervals was indicated.

**Table 1:** Difference tests of lane deviations in different distraction duration districts.

Factor	N	Subset for alpha = 0.05			
		1	2	3	4
[0.0,1.0)	30	9.50			
[1.0,1.5)	66	10.30			
[1.5,2.0)	56	11.70	11.70		
[2.0,2.5)	36		15.69		
[2.5,3.0)	21		15.95		
[3.0,3.5)	21			21.67	
[3.5,6.0)	17				36.76
Sig.		.408	.106	1.000	1.000

**Fig 7:**
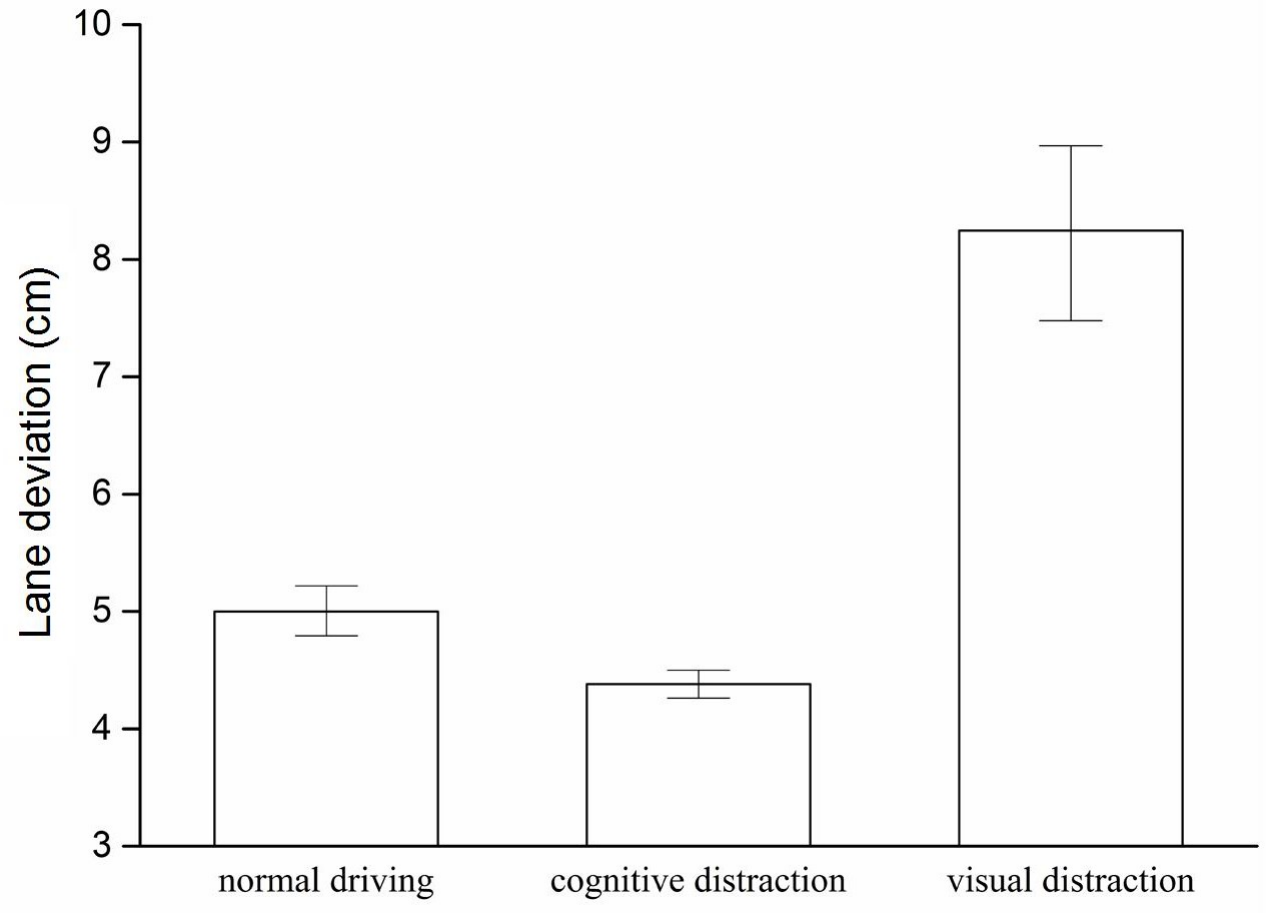
Comparison of lane deviation rates.

Ostlund et al. [32] found that lateral deviation increased as task duration increased. To eliminate this effect, the rate of lane deviation was selected while comparing the effects of visual distractions with those of cognitive distractions on lateral performance. The rates of lane deviation during cognitive distractions, visual distractions, and normal driving are presented in Fig 7.

One-way ANOVA was conducted on the rate of lane deviation in SPSS. The main effects of secondary tasks are listed in Table 2. Findings indicated that the distraction task significantly influenced the lateral driving performance of the driver (F(2, 6053) = 349.363, *p* < 0.001).

**Table 2:** Tests of between-subjects effects.

Source	Type III Sum of Squares	df	Mean Square	F	Sig.
Corrected Model	13173.986a	2	6586.993	349.363	.000
Intercept	168766.574	1	168766.574	8951.104	.000
Task	13173.986	2	6586.993	349.363	.000
Error	114124.929	6053	18.854		
Total	295860.067	6056			
Corrected Total	127298.916	6055			

Post-hoc test results are listed in Table 3. Compared with the rate of lane deviation during normal driving (5.00 cm), the rate of lane deviation during visual distraction (8.25 cm) is significantly higher; however, the cognitive distraction task significantly decreases the rate of lane deviation (4.38 cm). Moreover, the rate of lane deviation during visual distraction is higher than that during cognitive distractions—that is, twice as much as that during cognitive distractions. These findings indicate that lane deviation is faster and more likely to cause traffic accidents during visually distracted driving.

**Table 3:** Multiple Comparisons.

(I) Task	(J) Task	Mean Difference (I-J)	Std. Error	Sig.	95% Confidence Interval	
					Lower Bound	Upper Bound
Cognitive	Normal	-.6140*	.13639	.000	-.8814	-.3466
	Visual	-3.8615*	.14690	.000	-4.1495	-3.5736
Normal	Cognitive	.6140*	.13639	.000	.3466	.8814
	Visual	-3.2475*	.17104	.000	-3.5828	-2.9122
Visual	Cognitive	3.8615*	.14690	.000	3.5736	4.1495
	Normal	3.2475*	.17104	.000	2.9122	3.5828

Based on observed means. The error term is Mean Square (Error) = 18.854.

*. The mean difference is significant at the .05 level.

## 4. Discussion

In this paper, we investigated the effects of two types of low-perceived-risk secondary tasks on the lateral performance of the drivers. With an instrumented vehicle, a normal driving task, a visual distraction task, and a cognitive distraction task were conducted on a real highway. Lane deviation was used as an indicator to evaluate the lane-keeping ability of the drivers. In the cognitive distraction task, we evaluated lane deviations during conversation; in the visual distraction task, drivers had to identify and estimate information (i.e., speed and distance) about a rear vehicle.

As low-perceived-risk secondary tasks, these distraction tasks exerted significantly different effects on the lateral performance of each driver. Results revealed that visual distractions significantly increased the lane deviation rate, whereas cognitive distractions slightly decreased this rate. The higher the lane deviation rate, the poorer the lane-keeping ability of the driver. Such reduction could lead to increased likelihood of collision between the subject vehicle and a vehicle on the adjacent lane. Although both tasks were considered low-risk [19, 20], experimental results suggest that observing a rear vehicle during actual driving clearly impairs the lane-keeping ability of the drivers; thus, visual tasks are high-risk. This finding confirms that roadside billboards can lead to a high rate of traffic accidents [35] and further indicates that drivers tend to perceive high-risk visual tasks as low-risk tasks. Communicating with passengers did not impair the lane-keeping ability of the drivers. Consistent with previous experiments [36], the cognitive task slightly affected the driving performance but did not compromise the driving safety of the participants, suggesting that some cognitive tasks are relatively safe.

The differential effects of visual and cognitive tasks may be explained as follows. Drivers tended to focus on visual distractors during most visual distraction tasks, such as observing through the rearview mirror and billboards; however, during cognitive distractions, drivers mostly continued looking at the lane in front of them, which helped them estimate the position of the vehicle. Notably, the conversation task could enhance the lane-keeping ability of the drivers, which is consistent with the naturalistic research that found cell phone conversations to reduce crash risk [37, 38]. In the current study, conversations may help the driver remain alert, preventing the driver from performing other distraction tasks because the driver needs to pay more attention to the road ahead.

The second reason is that the drivers generally did not consider observing a rear vehicle to be dangerous; while performing this task, they were clearly focused on the rear car. Although the driver could perceive lane departure by the positional relationship between the lane marking in the rearview mirror [39], a lack of risk awareness led to a higher lane deviation rate. However, during cognitive distractions, the drivers did not need to look at an object; they could continue looking at the lane in front of them, which helped them adjust the position of the vehicle. Muhrer and Kaber [40, 41] indicated that the drivers must adjust their driving behavior in accordance with the driving environment ahead. However, while performing a visual distraction task, the driver could not observe the curvature of the upcoming road; thus, the relative lane position of the car could not be adjusted promptly.

Therefore, during driver training, the driver should be reminded of the danger of observing the surrounding environment for an extended period. Even when observing a rear vehicle through the rearview mirror, drivers should pay attention to lane keeping.

Moreover, the results of the visual distraction task revealed two findings. More than 90% of the drivers could observe the rearview mirror within 0.5–3.5 s, and more than half of the tasks could be completed within 1–2 s, consistent with a previous study [42].

The other notable finding is that lane deviations increased with an increase in distraction duration. This occurrence was similar to previous a finding [43], implying that distraction duration is positively correlated with the occurrence of an accident. Brumby and Salvucci [44, 45] found that lane deviation increased with phone-dialing duration while driving; this growing trend of lane deviation supports the results in the present study. Moreover, the current study indicates that the relationship between lane deviation and distraction duration was not a simple linear association; rather, the increasing trend of lane deviation with distraction duration could be categorized into three stages: 1) when the distraction duration was less than 2.0 s, the increase in lane deviation with distraction duration was not apparent; 2) when the distraction duration was 2.0–3.0 s, lane deviation increased gradually; and 3) when the distraction duration was longer than 3.0 s, lane deviation increased rapidly. This finding may be attributed to working memory capacity [46, 47]. For a short-term visual distraction, the driver still remembers the lane ahead while performing the distraction task, which can help the driver maintain safe driving temporarily. However, for a long-term visual distraction, the load of the distraction task exceeds the working memory capacity of the driver; the longer the distraction time, the greater the lane deviation rate. Ahlstrom and Zhang [48, 49] discussed the influence of a distraction warning algorithm on driver attention. These results can help improve distraction warning systems because a reasonable warning threshold can decrease false positives and improve driver acceptance of such systems.

A large number of factors, such as driving environment, weather, and vehicle model, may affect driving behavior. To improve the internal validity, all experiments were conducted under the same conditions. Moreover, to ensure external fidelity, participants were recruited from the entire population rather than limiting the recruitment to only students or teachers. Factors such as age and educational background are not controlled, thereby ensuring diversity of the subjects.

## 5. Conclusion

On the basis of an experiment on a real highway, this study presents a novel study on driving behaviors during low-perceived-risk secondary tasks. Moreover, this study compares the differences between the real risk and perceived risk of secondary tasks. The experiment findings emphasize that the perceived risk is not consistent with the actual risk of secondary tasks; in addition, drivers are inclined to underestimate the risk of visual distraction tasks. However, the actual risk of cognitive tasks at a low level of perceived risk is the same as the perceived risk of the driver. This study also demonstrates that lane deviation increases with distraction duration. These findings can be applied in the design of advanced driving-assistance systems and in improving professional driver training programs.

## 6. Limitations

This study has several limitations. The first limitation is that distraction duration cannot be further categorized because of insufficient experimental data. The effect of distraction duration on lateral vehicle deviation can be explored in greater detail in subsequent studies by using a shorter distraction time. In future research, we intend to further compare high-perceived-risk secondary tasks with low-perceived-risk secondary tasks.

The second limitation is that the sample size is relatively small. Possible differences in lane-keeping ability during distracted driving across gender and age groups were neither evaluated nor controlled. Previous studies have indicated that the gender and age of the driver may affect driving behavior [50, 51]. However, the current experiment was conducted on a real highway with potential risks, resulting in a smaller number of female and old-age applicants. To ensure the safety of the experiment, recruitment was restricted to participants with no accidents for the past three years. This constraint led to having an insufficient number of participants and recruiting mostly middle-aged men. Future studies intend to investigate the perceived risk and driving behavior between groups, including gender, age, and so on.
